# Mental health care in oncology. Contemporary perspective on the psychosocial burden of cancer and evidence-based interventions

**DOI:** 10.1017/S2045796019000866

**Published:** 2020-01-09

**Authors:** R. Caruso, W. Breitbart

**Affiliations:** 1 Department of Biomedical and Specialty Surgical Sciences, Institute of Psychiatry, University of Ferrara, Ferrara, Italy; 2 Department of Psychiatry & Behavioral Sciences, Jimmie C. Holland Chair in Psychiatric Oncology, Memorial Sloan Kettering Cancer Center, New York, NY, USA

**Keywords:** Cancer, cancer burden, consultation-liaison psychiatry, psychiatric disorders, psycho-oncology

## Abstract

With cancer incidence increasing over time worldwide, attention to the burden of psychiatric and psychosocial consequences of the disease is now mandatory for both cancer and mental health care professionals. Psychiatric disorders have been shown to affect at least 30–35% of cancer patients during all phases of the disease trajectory, and differ in nature according to stage and type of cancer. Other clinically relevant distressing psychosocial and existential conditions (e.g. demoralisation, health anxiety, loss of meaning and existential distress) not included as ‘disorders’ in the usual diagnostic and nosological systems (i.e. meta-diagnostic conditions) have also been shown to be present in another 15–20% of cancer patients. In this editorial, we will present a summary of the extensive literature regarding the epidemiology of the several psychosocial disorders affecting cancer patients as a cause of distress and burden to be taken into consideration and addressed in cancer care through evidence-based intervention.

## Introduction

Over the past 70 years, extensive research in oncology has demonstrated that cancer has significant psychosocial consequences for both the patients and the family in terms of psychiatric and psychosocial morbidity influencing the overall burden of the disease (Girgis *et al*., 2013; Caruso *et al*., 2017; Mehnert *et al.*, 2018). When the usual nosological systems of psychiatric classification (e.g. the International Classification of Disease – ICD, of the World Health Organization or the Diagnostic and Statistical Manual of Mental Disorders – DSM, of the American Psychiatric Association) are employed the rate of psychiatric morbidity is about one out of three patients (Mitchell *et al*., 2011). Data regarding cancer populations both in Northern (Mehnert *et al*., 2014; Kuhnt *et al*., 2016) and Southern Europe (Grassi *et al*., 2009; Grassi *et al*., 2018) confirm that 35–40% of cancer patients have a diagnosable psychiatric disorder according to the ICD-10 psychiatric interview. These figures are higher among cancer patients with advanced stages of cancer and in palliative care settings (Breitbart and Alici, 2014; Jaiswal *et al*., 2014), but not much lower among cancer survivors, in whom the burden of having had cancer has typically been expected to decrease over time. In fact, data show that about one-third of cancer survivors continue to suffer from psychiatric disorders (Geue *et al*., 2018) with a risk two to three times higher than control health groups and higher than the general population in terms of depressive and anxious symptomatology (Götze *et al*., 2019; Kuba *et al*., 2019).

## The characterisation of psychosocial morbidity in cancer

When examining the characteristic psychiatric diagnoses amongst cancer patients, some disorders are more common than others, such as depression, trauma/stress-related disorders and anxiety disorders. More recently, other conditions, including sub-threshold and clinically significant psychosocial/existential syndromes, not included as disorders, and thus not detectable by using ICD or DSM systems, have been identified as significant sources of distress and psychological burden for many cancer patients.

### Depressive spectrum disorders

Depressive spectrum disorders are extremely common in cancer, although their prevalence varies considerably depending on the studies, the treatment settings (outpatient clinics, hospital and palliative care settings), the stage of cancer (early diagnosis, recurrence, survivorship or advanced stages) and the type of assessment methods and tools used to determine caseness (e.g. self-report tools *v*. psychiatric interviews) (Walker *et al*., 2013; Krebber *et al.*, 2014; Caruso *et al*., 2017). With regards to major depression, the debate related to diagnostic problems has been very intense, because of the limits of a categorical approach and the need to understand the diagnostic specificity of physical *v*. psychological symptoms (affective *v.* neurovegetative) in the clinical manifestation of the disorder. For example, in palliative care, a marked reduction in performance, pain and fatigue, as well as loss of a vision of the future, make it difficult to differentiate major depression from other depressive spectrum disorders (Rayner *et al*., 2009; Wasteson *et al*., 2009; Fairman and Irwin, 2013; Janberidze *et al*., 2014). Clarification is also needed with respect to other forms of depression categorised in DSM-5 and ICD-10, such as minor depression, recurrent brief depression and anxious-depressive syndrome including sub-threshold and anxious-depressive, provided it is useful to use these categories in the context of cancer care (Mitchell *et al*., 2012).

### Anxiety disorders and trauma/stress-related disorders

Regarding anxiety disorders, specific phobias (in particular those related to well-known phenomena such as chemotherapy induced anticipatory nausea and vomiting, overlapping to the mechanisms of classic phobias) are common in cancer care, particularly after chemotherapy.

Trauma/stress-related disorders include post-traumatic stress disorder (PTSD) and sub-syndromic forms of PTSD hat has also shown to affect 15% of cancer patients (Cordova *et al*., 2017) with a smaller percentage, but still significant, in long-survivors of cancer [at least 5 years after diagnosis (Rustad *et al*., 2012)]. These data should be taken into consideration when analysing some aspects of PTSD in cancer diseases, such as the impossibility to define qualitative (exogenous stressors *v.* endogenous stressors) and temporal criteria (acute stressor in PTSD *v.* continuing and multiple stressful events in cancer).

Adjustment disorders (ADs) represent a frequent diagnosis at all stages of cancer, and describe the emotional reactions related to the multiple stressors linked to cancer, involving 20–25% of patients. Anxiety as well as depression can be also components of AD depression with intertwining effects in the manifestation of both conditions. However, several different problems emerge, not only with regards to oncology but all medical conditions, because of the low specificity of DSM criteria for the diagnosis of AD, the vagueness of the symptoms, the weakness of the concept of distress *v*. the expected normal response, and the inconsistency and inadequacy of using time as a criterion (Casey, 2014; Bachem and Casey, 2018).

### Somatisation and somatic-symptom disorders

Somatisation and somatic-symptom disorders and their role in influencing patients' well-being are also important, not only because of its prevalence especially in long-survivors of cancer, but also in general for the multiple relationships between the perception of somatic symptoms and the patient's interpretation. Pain intensity, hypochondriacal concerns, disease labelling and dysfunctional interpretation of somatic symptoms, are part of a complex picture related to illness and abnormal illness behaviour; ways in which individuals adaptively or maladaptively monitor their body, experience, evaluate and respond to their state of health. All these issues have a significant role in cancer care, since somatic symptoms may magnify disability resulting from cancer, interfere with treatment adherence, decision making, cause delay in recovery, result in poor outcomes and recurrence, and reduce overall well-being and quality of life, and complicate the diagnosis of major depression (due to the overlap of symptoms occurring as a result of the underlying disease, depression or somatoform disorders) (Chaturvedi *et al*., 2006; Grassi *et al*., 2013).

### Neurocognitive disorders

Neurocognitive disorders represent an important clinical area in terms of mild (sometimes major) cognitive impairment or acute confusional states (delirium) secondary to the disease and its treatment. Mild neurocognitive disorders can be secondary to chemotherapy or chemotherapy associated with radiation therapy which can cause impairment of neuropsychological domains, such as memory, attention, concentration, learning functions, calculation and visual-spatial perception (Wigmore, 2013). Other cognitive impairment conditions, including dementia (especially in patients undergoing radiotherapy of the brain for the treatment of brain metastases) are estimated to be 2–5%. They could be caused by primary disease (e.g. primary brain tumours) or by the consequences of cancer treatments on the central nervous system (CNS). Brain metastases can also induce psychopathological disorders, in particular memory impairment associated with personality modification, as well as aphasia, alexia, acalculia, agnosia, apraxia, amnesia and mood disorders (Soussain *et al*., 2009).

A special area of interest is represented by delirium, one of the most common cognitive disorders in cancer, particularly in the advanced stages of disease. Delirium prevalence, depending on stage of disease, ranges from 10 to 80%, including both the hyperactive-agitated and mixed forms, although hypoactive delirium is most common in advanced cancer (Stagno *et al*., 2004; Breitbart and Alici, 2012, 2014; Grassi *et al*., 2015; Hey *et al*., 2015; Lawlor and Bush, 2015).

### Other psychosocial clinically significant disorders

Apart from the aforesaid psychiatric disorders, as emerging from the DSM and the ICD nosography, other significant clinical conditions have been shown to negatively affect the patient's quality of life. Research based on the use of the Diagnostic Criteria for Psychosomatic Research as well as other tools has indicated the importance of assessing a series of dimensions and emotional responses, such as demoralisation, loss of meaning, existential distress, irritable mood, health anxiety and illness denial that can all be of impact in oncology patients. Health anxiety (37.7%) and demoralisation (28.8%) are for example the most frequent clinically relevant psychosocial conditions diagnosable in cancer patients in different phases of illness (Grassi *et al*., 2005). Demoralisation, as a syndrome characterised by helplessness, hopelessness, a sense of failure and the inability to cope, has been shown to be associated with higher levels of sadness, more physical symptoms, poorer well-being, poorer leisure activity and lower support from interpersonal ties, higher levels of worries and preoccupation related to cancer (e.g. the illness itself, the effects of treatment, feeling different from others, the impact on sexual life, the future) as well as loss of meaning and hope, poor dignity, a sense of worthlessness on one's own life and in the future and suicidal ideation (Grassi and Nanni, 2016).

### Sexual disorders and dysfunctions

Sexual disorders and dysfunctions are also part of the possible psychiatric diagnoses in cancer involving an average of 25–40% of cancer patients, both in male and female cancer patients (Morreale, 2011). Patients with breast cancer, ovary cancer and uterine cancer show a decrease or loss of sexual drive, changes in female genital response (decrease or loss of lubrication), orgasm problems, and vaginismus and dyspareunia. Similarly, male patients show that testicular and prostate cancer have shown to report important consequences on sexuality, in particular low sexual drive, performance anxiety, premature ejaculation, erectile dysfunction and inhibition of orgasm (Chung and Brock, 2013; Chung and Gillman, 2014; Katz and Dizon, 2016). Body image changes, the effects of chemo- and radiation therapy at a systemic level as well as on the reproductive tract; weight loss, stomas (e.g. ostomy and colostomy), the consequences of surgery and incontinence are the main causal factors of these disorders.

## Evidenced-based psychiatric and psycho-social interventions

Knowledge about the treatment of psychosocial and psychiatric conditions is mandatory for cancer care clinicians, given the number of studies that have shown the efficacy and effectiveness of psychosocial interventions (Faller *et al*., 2013; Myrhaug *et al*., 2018). Counselling, educational, coping and psychological support, and more specific forms of psychotherapy in their different formats (group, individual and family therapy) and orientations (cognitive-behavioural, supportive-expressive, existential, meaning-centred and psychodynamic) have been developed for cancer patients in order to more specifically intervene in all the conditions where psychological disorders and maladjustment to cancer and treatment emerge. The choice of intervention is related to several variables, including the clinical psychological condition, the type and phase of illness, the context, as well as the availability of psycho-oncology services with trained professionals which should be part of multidisciplinary teams.

The literature on the efficacy of the several forms of specialised psychotherapeutic interventions and psychosocial rehabilitation, especially if in the form of collaborative care, indicates a general benefit in reducing the severity of psychiatric symptoms, as well as somatic symptoms (e.g. pain) and in improving quality of life, well-being and return to work and illness behaviour (Li *et al*., 2017). The interventions, with the most empirical support for treating distress in cancer patients include supportive-expressive group psychotherapy, cognitive-behavioural and cognitive-existential therapy, and meaning centred psychotherapy (Breitbart, 2017; Kang *et al*., 2019)

Integrated psychopharmacological intervention (*psychopharm-oncology*) (Grassi and Riba, 2014) has also shown to be efficacious in several disorders, where the use of specific serotonin reuptake inhibitors; specific noradrenergic and serotonin reuptake inhibitors have been shown to help both in treating depression and anxiety and cancer-related symptoms, such as pain, hot-flashes and pain (Caruso *et al*., 2013; Grassi *et al*., 2014). With respect to this it is important for clinicians, usually psychiatrists, but also oncologists and primary care physicians to have a proper training on the use of the drugs, their side-effects and interaction with other cancer treatment in oncology (Grassi *et al*., 2018). There is however an urgent need for a systemic approach to the development and conduction of multidisciplinary integrated psychosocial interventions, based on both guidelines and larger and more rigorously conducted randomised controlled trials.

## Conclusions

Cancer has significant psychosocial consequences for both patients and their families. Today there is scientific evidence of the benefits of providing psychosocial cancer care as part of standard care in reducing distress and psychosocial morbidity associated with cancer and in fostering a better quality of life during and after treatment, and eventually in increasing survival. The significant advances in research in the area of psycho-oncology have favoured the development, implementation and dissemination of psychosocial guidelines and evidence-based treatments for several co-morbid psychiatric disorders in cancer, such as depression (Li *et al*., 2017) and anxiety (Traeger *et al*., 2012). Screening, identification and access to evidence-based psychosocial approaches for cancer patients in distress must be provided, both in the hospital and in community settings (Lazenby, 2015; Andersen and Dorfman, 2016; Grassi *et al.,* 2018). Besides specific cancer care specialists, primary care should also take responsibility in its role of provision of continuous, coordinated and comprehensive care for individuals with cancer and families. This should include psychosocial care, prevention and diagnosis, in shared follow-up and survivorship care and end-of-life care (Rubin *et al*., 2015).

## References

[ref1] Andersen BL and Dorfman CS (2016) Evidence-based psychosocial treatment in the community: considerations for dissemination and implementation. Psycho-Oncology 25, 482–490. 27092813 10.1002/pon.3864 PMC4839194

[ref2] Bachem R and Casey P (2018) Adjustment disorder: a diagnosis whose time has come. Journal of Affective Disorders 227, 243–225. 29107817 10.1016/j.jad.2017.10.034

[ref3] Breitbart W (ed.) (2017) Meaning-Centered Psychotherapy in the Cancer Setting: Finding Meaning and Hope in the Face of Suffering. New York: Oxford University Press.

[ref4] Breitbart W and Alici Y (2012) Evidence-based treatment of delirium in patients with cancer. Journal of Clinical Oncology 30, 1206–1214. 22412123 10.1200/JCO.2011.39.8784 PMC3646320

[ref5] Breitbart W and Alici Y (2014) Psychosocial Palliative Care. New York: Oxford University Press.

[ref6] Caruso R, Grassi L, Nanni MG and Riba M (2013) Psychopharmacology in psycho-oncology. Current Psychiatry Reports 15, 393. 23949568 10.1007/s11920-013-0393-0

[ref7] Caruso R, Nanni MG, Riba MB, Sabato S and Grassi L (2017) The burden of psychosocial morbidity related to cancer: patient and family issues. International Review of Psychiatry 29, 389–402. 28753076 10.1080/09540261.2017.1288090

[ref8] Casey P (2014) Adjustment disorder: new developments. Current Psychiatry Reports 16, 451. 24748555 10.1007/s11920-014-0451-2

[ref9] Chaturvedi SK, Maguire P and Somashekar BS (2006) Somatization in cancer. International Review of Psychiatry 18, 49–54. 16451880 10.1080/09540260500466881

[ref10] Chung E and Brock G (2013) Sexual rehabilitation and cancer survivorship: a state of art review of current literature and management strategies in male sexual dysfunction among prostate cancer survivors. The Journal of Sexual Medicine 10(suppl. 1), 102–111. 23387915 10.1111/j.1743-6109.2012.03005.x

[ref11] Chung E and Gillman M (2014) Prostate cancer survivorship: a review of erectile dysfunction and penile rehabilitation after prostate cancer therapy. Medical Journal of Australia 200, 582–585. 24882489 10.5694/mja13.11028

[ref12] Cordova MJ, Riba MB and Spiegel D (2017) Post-traumatic stress disorder and cancer. The Lancet. Psychiatry 4, 330–338. 28109647 10.1016/S2215-0366(17)30014-7 PMC5676567

[ref13] Fairman N and Irwin SA (2013) Palliative care psychiatry: update on an emerging dimension of psychiatric practice. Current Psychiatry Reports 15, 374. 23794027 10.1007/s11920-013-0374-3 PMC3762470

[ref14] Faller H, Schuler M, Richard M, Heckl U, Weis J and Küffner R (2013) Effects of psycho-oncologic interventions on emotional distress and quality of life in adult patients with cancer: systematic review and meta-analysis. Journal of Clinical Oncology 31, 782–793. 23319686 10.1200/JCO.2011.40.8922

[ref15] Geue K, Brähler E, Faller H, Härter M, Schulz H, Weis J, Koch U, Wittchen HU and Mehnert A (2018) Prevalence of mental disorders and psychosocial distress in German adolescent and young adult cancer patients (AYA). Psycho-Oncology 27, 1802–1809. 29644783 10.1002/pon.4730

[ref16] Girgis A, Lambert S, Johnson C, Waller A and Currow D (2013) Physical, psychosocial, relationship, and economic burden of caring for people with cancer: a review. Journal of Oncology Practice 9, 197–202. 23942921 10.1200/JOP.2012.000690 PMC3710169

[ref17] Götze H, Friedrich M, Taubenheim S, Dietz A, Lordick F and Mehnert A (2019) Depression and anxiety in long-term survivors 5 and 10 years after cancer diagnosis. Supportive Care in Cancer 28, 211–220. 31001695 10.1007/s00520-019-04805-1

[ref20a] Grassi L and Nanni MG (2016) Demoralization syndrome: New insights in psychosocial cancer care. Cancer 122, 2130–2133. 27171755 10.1002/cncr.30022

[ref18] Grassi L and Riba M (eds) (2014) Psychopharmacology in Oncology and Palliative Care. Berlin/New York: Springer.

[ref19] Grassi L, Sabato S, Rossi E, Marmai L and Biancosino B (2009) Affective syndromes and their screening in cancer patients with early and stable disease: Italian ICD-10 data and performance of the distress thermometer from the Southern European Psycho-Oncology Study (SEPOS). Journal of Affective Disorders 114, 193–199. 18757101 10.1016/j.jad.2008.07.016

[ref20] Grassi L, Sabato S, Rossi E, Biancosino B and Marmai L (2005) Use of the diagnostic criteria for psychosomatic research in oncology. Psychotherapy and Psychosomatics 74, 100–107. 15741759 10.1159/000083168

[ref21] Grassi L, Caruso R and Nanni MG (2013) Somatization and somatic symptom presentation in cancer: a neglected area. International Review of Psychiatry 25, 41–51. 23383666 10.3109/09540261.2012.731384

[ref22] Grassi L, Caruso R, Hammelef K, Nanni MG and Riba M (2014) Efficacy and safety of pharmacotherapy in cancer-related psychiatric disorders across the trajectory of cancer care: a review. International Review of Psychiatry 26, 44–62. 24716500 10.3109/09540261.2013.842542

[ref23] Grassi L, Caraceni A, Mitchell AJ, Nanni MG, Berardi MA, Caruso R and Riba M (2015) Management of delirium in palliative care: a review. Current Psychiatry Reports 17, 550. 25663153 10.1007/s11920-015-0550-8

[ref24] Grassi L, Caruso R, Mitchell AJ, Sabato S and Nanni MG (2018) Screening for emotional disorders in patients with cancer using the Brief Symptom Inventory (BSI) and the BSI-18 versus a standardized psychiatric interview (the World Health Organization Composite International Diagnostic Interview). Cancer 124, 2415–2242. 29660109 10.1002/cncr.31340

[ref25] Grassi L, Nanni MG, Rodin G, Li M and Caruso R (2018) The use of antidepressants in oncology: a review and practical tips for oncologists. Annals of Oncology 29, 101–111. 29272358 10.1093/annonc/mdx526

[ref26] Hey J, Hosker C, Ward J, Kite S and Speechley H (2015) Delirium in palliative care: detection, documentation and management in three settings. Palliative & Supportive Care 13, 1541–1545. 24139058 10.1017/S1478951513000813

[ref27] Jaiswal R, Alici Y and Breitbart W (2014) A comprehensive review of palliative care in patients with cancer. International Review of Psychiatry 26, 87–101. 24716503 10.3109/09540261.2013.868788

[ref28] Janberidze E, Hjermstad MJ, Haugen DF, Sigurdardottir KR, Løhre ET, Lie HC, Kaasa S, Knudsen AK and EURO IMPACT (2014) How are patient populations characterized in studies investigating depression in advanced cancer? Results from a systematic literature review. Journal of Pain and Symptom Management 48, 678–698. 24681108 10.1016/j.jpainsymman.2013.11.013

[ref29] Kang KA, Han SJ, Lim YS and Kim SJ (2019) Meaning-centered interventions for patients with advanced or terminal cancer: a meta-analysis. Cancer Nursing 42, 332–340. 30024439 10.1097/NCC.0000000000000628

[ref30] Katz A and Dizon DS (2016) Sexuality after cancer: a model for male survivors. Journal of Sexual Medicine 13, 70–78. 26755089 10.1016/j.jsxm.2015.11.006

[ref31] Krebber AM, Buffart LM, Kleijn G, Riepma IC, de Bree R, Leemans CR, Becker A, Brug J, van Straten A, Cuijpers P and Verdonck-de Leeuw IM (2014) Prevalence of depression in cancer patients: a meta-analysis of diagnostic interviews and self-report instruments. Psycho-Oncology 23, 121–130. 24105788 10.1002/pon.3409 PMC4282549

[ref32] Kuba K, Esser P, Mehnert A, Hinz A, Johansen C, Lordick F and Götze H (2019) Risk for depression and anxiety in long-term survivors of hematologic cancer. Health Psychology 38, 187–195. 30762398 10.1037/hea0000713

[ref33] Kuhnt S, Brähler E, Faller H, Härter M, Keller M, Schulz H, Wegscheider K, Weis J, Boehncke A, Hund B, Reuter K, Richard M, Sehner S, Wittchen HU, Koch U and Mehnert A (2016) Twelve-month and lifetime prevalence of mental disorders in cancer patients. Psychotherapy and Psychosomatics 85, 289–296. 27508418 10.1159/000446991

[ref34] Lawlor PG and Bush SH (2015) Delirium in patients with cancer: assessment, impact, mechanisms and management. Nature Reviews Clinical Oncology 12, 77–92. 10.1038/nrclinonc.2014.147 25178632

[ref35] Lazenby M, Tan H, Pasacreta N, Ercolano E and McCorkle R (2015) The five steps of comprehensive psychosocial distress screening. Current Oncology Reports 17, 447. 25824699 10.1007/s11912-015-0447-z PMC4918509

[ref36] Li M, Kennedy EB, Byrne N, Gérin-Lajoie C, Katz MR, Keshavarz H, Sellick S and Green E (2017) Systematic review and meta-analysis of collaborative care interventions or depression in patients with cancer. Psycho-Oncology 26, 573–587. 27643388 10.1002/pon.4286

[ref37] Mehnert A, Brähler E, Faller H, Härter M, Keller M, Schulz H, Wegscheider K, Weis J, Boehncke A, Hund B, Reuter K, Richard M, Sehner S, Sommerfeldt S, Szalai C, Wittchen HU and Koch U (2014) Four-week prevalence of mental disorders in patients with cancer across major tumor entities. Journal of Clinical Oncology 32, 3540–3546. 25287821 10.1200/JCO.2014.56.0086

[ref38] Mehnert A, Hartung TJ, Friedrich M, Vehling S, Brähler E, Härter M, Keller M, Schulz H, Wegscheider K, Weis J, Koch U and Faller H (2018) One in two cancer patients is significantly distressed: prevalence and indicators of distress. Psycho-Oncology 27, 75–82. 28568377 10.1002/pon.4464

[ref39] Mitchell AJ, Chan M, Bhatti H, Halton M, Grassi L, Johansen C and Meader N (2011) Prevalence of depression, anxiety, and adjustment disorder in oncological, haematological, and palliative-care settings: a meta-analysis. The Lancet Oncology 12, 160–174. 21251875 10.1016/S1470-2045(11)70002-X

[ref40] Mitchell AJ, Meader N, Davies E, Clover C, Crater G, Loscalzo M, Linden W, Grassi L, Johansen C, Carlson L and Zabora J (2012) Meta-analysis of screening and case finding tools for depression in cancer: evidence based recommendations for clinical practice on behalf of the DCC Consensus Group. Journal of Affective Disorders 140, 149–160. 22633127 10.1016/j.jad.2011.12.043

[ref41] Morreale MK (2011) The impact of cancer on sexual function. Advances in Psychosomatic Medicine 31, 72–82. 22005205 10.1159/000328809

[ref42] Myrhaug HT, Mbalilaki JA, Lie NK, Hansen T and Nordvik JE (2018) The effects of multidisciplinary psychosocial interventions on adult cancer patients: a systematic review and meta-analysis. Disability and Rehabilitation 29, 1–9. 10.1080/09638288.2018.1515265 30497305

[ref43] Rayner L, Loge JH, Wasteson E, Higginson IJ and EPCRC, European Palliative Care Research Collaborative (2009) The detection of depression in palliative care. Current Opinion in Supportive and Palliative Care 3, 55–56 19365162 10.1097/SPC.0b013e328326b59b

[ref44] Rubin G, Berendsen A, Crawford SM, Dommett R, Earle C, Emery J, Fahey T, Grassi L, Grunfeld E, Gupta S, Hamilton W, Hiom S, Hunter D, Lyratzopoulos G, Macleod U, Mason R, Mitchell G, Neal RD, Peake M, Roland M, Seifert B, Sisler J, Sussman J, Taplin S, Vedsted P, Voruganti T, Walter F, Wardle J, Watson E, Weller D, Wender R, Whelan J, Whitlock J, Wilkinson C, de Wit N and Zimmermann C (2015) The expanding role of primary care in cancer control. The Lancet Oncology 16, 1231–1272. 26431866 10.1016/S1470-2045(15)00205-3

[ref45] Rustad JK, David D and Currier MB (2012) Cancer and post-traumatic stress disorder: diagnosis, pathogenesis and treatment considerations. Palliative and Supportive Care 10, 213–223. 22436138 10.1017/S1478951511000897

[ref46] Soussain C, Ricard D, Fike JR, Mazeron JJ, Psimaras D and Delattre JY (2009) CNS Complications of radiotherapy and chemotherapy. Lancet 374, 1639–1651. 19897130 10.1016/S0140-6736(09)61299-X

[ref47] Stagno D, Gibson C and Breitbart W (2004) The delirium subtypes: a review of prevalence, phenomenology, pathophysiology, and treatment response. Palliative and Supportive Care 2, 171–179. 16594247 10.1017/s1478951504040234

[ref48a] Traeger L, Greer JA, Fernandez-Robles C, Temel JS and Pirl WF (2012) Evidence-based treatment of anxiety in patients with cancer. Journal of Clinical Oncology 30, 1197–1205. 22412135 10.1200/JCO.2011.39.5632

[ref48] Walker J, Holm Hansen C, Martin P, Sawhney A, Thekkumpurath P, Beale C, Symeonides S, Wall L, Murray G and Sharpe M (2013) Prevalence of depression in adults with cancer: a systematic review. Annals of Oncology 24, 895–900. 23175625 10.1093/annonc/mds575

[ref49] Wasteson E, Brenne E, Higginson IJ, Hotopf M, Lloyd-Williams M, Kaasa S, Loge JH and European Palliative Care Research Collaborative (EPCRC) (2009) Depression assessment and classification in palliative cancer patients: a systematic literature review. Palliative Medicine 23, 739–753 19825894 10.1177/0269216309106978

[ref50] Wigmore P (2013) The effect of systemic chemotherapy on neurogenesis, plasticity and memory. Current Topics in Behavioral Neurosciences 15, 211–240. 23239468 10.1007/7854_2012_235

